# Case report of *Triumeq* (abacavir/dolutegravir/lamivudine) associated rhabdomyolysis in a human immunodeficiency virus (HIV) infected patient

**DOI:** 10.1097/MD.0000000000015149

**Published:** 2019-04-26

**Authors:** Muhammad Saad, Fernando Casado-Castillo, Paul Kelly

**Affiliations:** Department of Internal Medicine, BronxCare Hospital Center, Bronx, New York.

**Keywords:** HIV infection, rhabdomyolysis, *Triumeq*

## Abstract

**Rationale:**

With the existence of the human immunodeficiency virus (HIV) infection as a chronic disease, more often adverse effects of its treatment with the various antiretroviral therapies (ARTs) available have been recognized. Going further, *Triumeq* has been associated with a myriad of adverse effects, of which rhabdomyolysis is rarely reported in the literature.

**Patient concerns:**

The patient presented with muscle tenderness over the lower limbs and dark brown-to-red colored urine.

**Diagnosis:**

Given the presenting symptoms, as well as the laboratory testing, including elevated serum creatine kinase and liver enzymes, the diagnosis of rhabdomyolysis was made.

**Interventions:**

Improvement was achieved rapidly after starting intravenous fluid therapy and with discontinuation of *Triumeq*.

**Outcomes:**

After discharge, repeated creatine kinase levels in the clinic have been normal and decision was made to initiate another ART and until now, no further episodes of rhabdomyolysis have developed. Regular outpatient follow-up has been ongoing for over 1 year and no complications have been identified.

**Lessons:**

This case aims to recognize rhabdomyolysis as a rare, but possible adverse effect associated with the use of *Triumeq* for HIV-infected patients and therefore clinicians prescribing this combination should be aware of this potential side effect and counsel their patients accordingly.

## 1 Introduction

Rhabdomyolysis is a complex medical condition involving the rapid dissolution of damaged or injured skeletal muscle. This disruption of skeletal muscle integrity leads to the direct release of intracellular muscle components, including myoglobin, creatine kinase (CK), aldolase and lactate dehydrogenase, as well as electrolytes, into the bloodstream and extracellular space.^[1,2]^ Acute rhabdomyolysis has been described during primary HIV infection, and also as an adverse reaction to certain drugs, including antiretroviral therapy (ART).^[3]^ At this time, we present an interesting case of rhabdomyolysis suspected to be associated with *Triumeq*, a side effect rarely described while receiving this ART combination.

## 2 Case presentation

A 57-year-old Hispanic male with a medical history of human immunodeficiency virus (HIV) infection, hepatitis C virus (HCV) infection, HIV related neuropathy and chronic degenerative disease of lumbar spine presented to the emergency room (ER) complaining of dark brown-to-red colored urine and bilateral lower extremity and lower back pain for 2 days before his presentation. He was in his normal state of health before the onset of symptoms. The patient denied any fever, chills, shortness of breath, chest pain, dizziness, recent trauma or immobilizations and there was no seizure activity or extreme exertion reported. He never had these symptoms before. His home medications included dolutegravir-abacavir-lamivudine (*Triumeq)* and omeprazole 40 mg taken occasionally for stomach discomfort. *Triumeq* was started 2 months prior to his presentation. He was allergic to penicillin. He was a former illicit drug user and former smoker, having stopped both habits more than 20 years ago since date of presentation. Review of systems was pertinent only for dark urine, lower back pain and bilateral lower limbs pain. Initial vital signs showed a temperature of 98.4°F (36.8°C), blood pressure 142/89 mm Hg, pulse rate 109 beats per minutes and respiratory rate 16 breath per minute, with a pulse oximetry saturation of 97% while breathing on room air; body mass index (BMI) was 21.8 kg/m^2^. Physical examination showed a cachectic male, with a warm and dry skin, conjunctiva was clear. Head and neck exam did not show any relevant findings. Lungs were clear to auscultation. Heart examination showed a regular rhythm, S1 and S2 were present; no murmurs or rub were noted. Abdomen was soft, nontender and nondistended; no organ distention or palpable masses were noted. Musculoskeletal examination revealed tenderness of lumbar spine and both lower extremities, with no signs of trauma. Neurological examination was grossly intact.

Initial investigations included a computerized tomography (CT) of the abdomen and pelvis without contrast material, which was grossly unremarkable. Initial laboratories are shown in Table 1. Significant analysis included a complete blood count with a red blood cell (RBC) of 4.32 MIL/μI, Hemoglobin was 16.1 g/dL, Hematocrit 47.5%, white blood cell (WBC) 14,000/μI. Basic metabolic profile showed a Potassium of 4.3 mEq/L, blood urea nitrogen (BUN) 30 mg/dL, Creatinine 1.2 mg/dL, estimated glomerular filtration rate (e-GFR) was 49 mlLmin/1.73m^2^. Liver function test showed an Aspartate Transaminase (AST) of 2,270 units/L, alanine transaminase (ALT) of 342 units/L and total bilirubin was within normal limits. Serum CK was 98,061 units/L (normal 20–200 units/L). Lactic acid was 2.3 mg/dL. Blood ethanol was <10. Urine toxicology screen was negative for recreational drugs. Urine analysis was red and turbid on gross examination; specific gravity was 1.030. Urine protein was more than 300 mg/dL, bilirubin was moderate elevated and blood was reported as large. Nitrates were positive and microscopy showed many bacteria and few mucous casts. Crystal examination revealed few amorphous phosphate crystals and pathological casts were significant for few fine granular casts. Blood and urine cultures were negative. The human leucocyte antigen (HLA)-B5701 was negative. His CD4+ cell count was 816 cell/microliter and performed HIV polymerase chain reaction (PCR) showed an undetectable viral load.

**Table 1:**
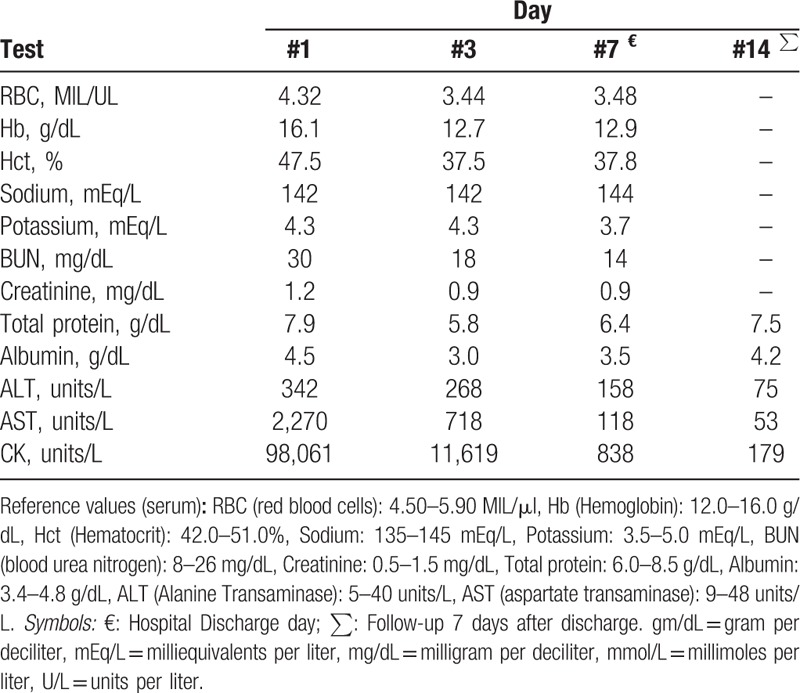
Laboratory results.

**Figure 1:**
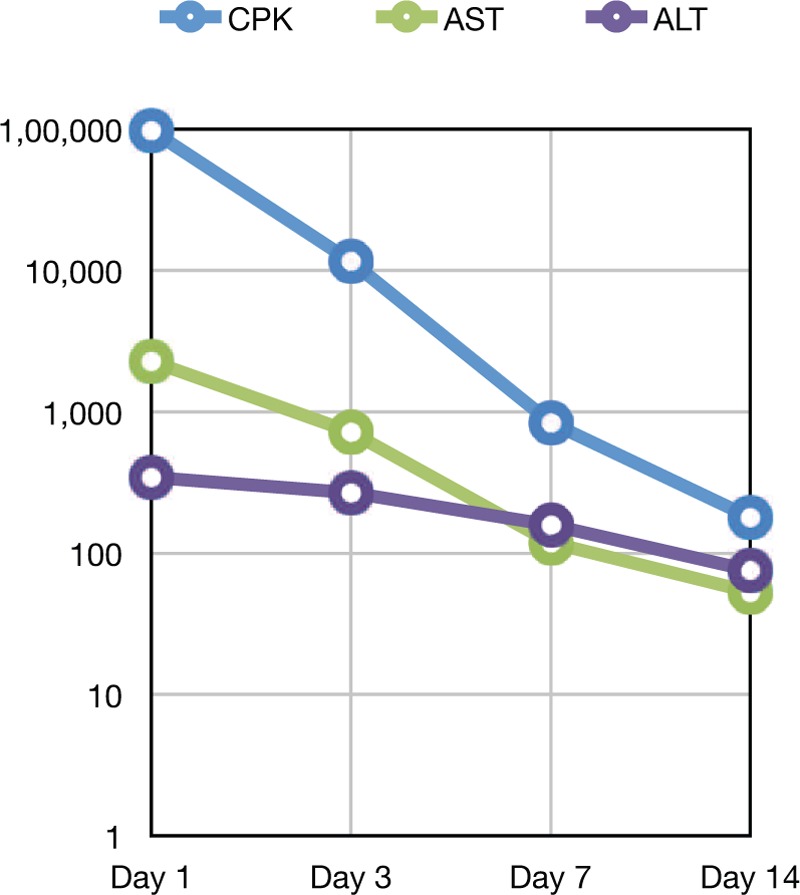
CPK and LFT trend. ALT = alanine transaminase, AST = aspartate transaminase, CPK = creatine phosphokinase, LFT = liver function test.

Given the severe rhabdomyolysis, the patient was started on high infusion rate intravenous fluid therapy with sodium chloride 0.9%. He was admitted to the intensive care unit (ICU) for further management; dolutegravir-abacavir-lamivudine (*Triumeq*) was held in view of severe rhabdomyolysis. In the ICU, the patient remained stable and intravenous fluid therapy was continued. His CK levels trended down from 98,061 units/L to 10,000 units/L on day 3 (see Table 1 and Fig. 1) and continued to decrease to 838 units/L on the day of discharge (hospital day 7). Follow-up in the clinic 1 week after discharge revealed that patient symptoms improved significantly and repeated CK levels were 179 units/L. In subsequent clinic visits, the patient was started on emtricitabine, elvitegravir, tenofovir, and cobicistat (*Genvoya*) and he remained stable with no further episodes of rhabdomyolysis noted. The Naranjo Scale^[4]^ (see Table 2) (Adverse Drug Reaction Probability Scale, developed to help standardize assessment of causality for all adverse drug reactions) score was 6 in our patient (1 point for previous conclusive reports of this reaction, 2 points for the adverse event appearing after the suspected drug was administered, 1 point for improvement after the drug was discontinued, 2 points for no alternatives causes that could on their own have caused the reaction).

**Table 2:**
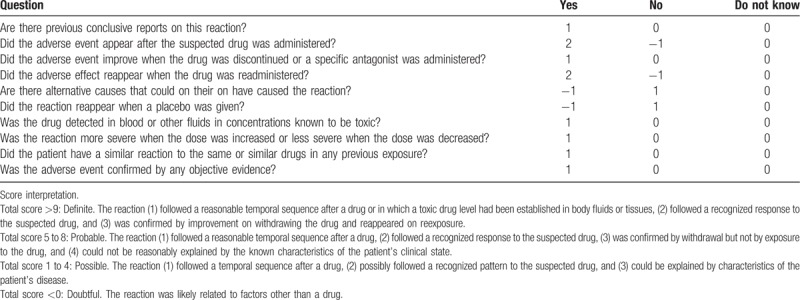
Naranjo scale^[4]^.

## 3 Discussion

Rhabdomyolysis is a syndrome characterized by muscle necrosis and release of intracellular muscle constituents into the circulation. CK levels are typically markedly elevated, and muscle pain with myoglobinuria may be present. The severity of illness ranges from asymptomatic elevations in serum muscle enzymes to life-threatening disease associated with extreme enzyme elevations, electrolyte imbalances, and acute kidney injury.^[1,2]^ Serum CK levels at presentation are usually at least 5 times the upper limit of normal, but range from ∼1500 to 100,000 international units/L. The mean peak CK reported for each of a variety of different causes and for patients with both single and multiple causes ranged from ∼10,000 to 25,000 in the largest series.^[2,3]^

Rhabdomyolysis is common in the HIV positive population, particularly in those with advanced disease.^[3,5]^ Risk factors unique to this population includes HIV infection itself, opportunistic infections, co-infection with HCV, medication-related adverse effects (including pentamidine, trimethoprim-sulfamethoxazole, sulfadiazine, and antiretroviral agents, such as zidovudine, raltegravir and abacavir), drug–drug interactions, malignancy, alcohol and/or illicit drug abuse.^[3,6,7]^ While infection is the most frequently encountered etiology of rhabdomyolysis among the HIV positive population, with sepsis being identified as major cause, Koubar et al demonstrated that 6% of cases of rhabdomyolysis in patients with HIV infection could be attributed to medications.^[5,8]^ Of the listed risk factors, our patient had co-infection with HCV and how it contributed to his presentation is unknown to us, but our suspicion of HCV as a major causative of rhabdomyolysis in our case was low, given the fact that after discontinuation of the offending drug, he did not developed any further episodes of rhabdomyolysis.

*Triumeq* is a combination of abacavir, dolutegravir and lamivudine, initially approved by the US Food and Drug Administration (FDA) in 2014 and is promoted as an effective, generally well tolerated once-daily fixed-dose tablet option for the management of HIV infection. Lamivudine is a nucleotide reverse transcriptase inhibitor, present in the market since 1995 in the form of other combinations and is comparatively well studied in terms of its adverse effect profile; It can cause allergic reaction, nausea, vomiting, diarrhea, lactic acidosis, and rarely rhabdomyolysis due to mitochondrial toxicity.^[9,10]^ There have been few cases reported in the literature regarding lamivudine induced rhabdomyolysis. Baharin et al^[11]^ reported a case of rhabdomyolysis associated to lamivudine administration in acute viral hepatitis B infection, which resolved after fluid therapy and 2 weeks of drug cessation. The suspicion for this agent as culprit for the development of rhabdomyolysis in our patient is low given mildly elevated lactic acid level on presentation, which has been associated as well to be elevated in the case of lamivudine associated rhabdomyolysis.

Dolutegravir is an integrase inhibitor FDA approved in 2013 with a range of adverse effects including liver enzyme abnormalities and more recently neuropsychiatric effects and weight gain^[12,13]^ however, to date there is no literature report of acute rhabdomyolysis with dolutegravir. Further investigation into the literature regarding adverse effects of myopathy or rhabdomyolysis derived from other integrase inhibitors (INSTI), reveals sporadic reports of muscle toxicity associated with raltegravir therapy and CK elevation of >200U/L in approximately 8.9% of patients without clinical signs of myopathy.^[14]^ Although the FDA has advised checking a CK level before initiation of raltegravir,^[15]^ no cases have been reported regarding treatment cessation secondary to rhabdomyolysis. The mechanism for muscle toxicity induced by raltegravir can be explained by either elevation of its level due to impaired hepatic glucuronidation associated with liver dysfunction or elevated levels owing to kidney failure.^[14]^ Whether it is a class effect is not known since to the best of our knowledge no cases have been reported with dolutegravir in the literature and hence its mechanism of muscle injury remains unclear. Another hypothesis proposed is variable pharmacodynamics of dolutegravir with other component drugs in *Triumeq* which could lead to an exaggerated dolutegravir muscle effect. Our patient did not have any classically associated risk factors for rhabdomyolysis such as alcohol use, antipsychotics, strenuous physical activity or underlying renal impairment. Liver enzymes elevation in our patient was suspected to be as a direct result of rhabdomyolysis, however drug toxicity may have also played a role, as literature reports elevation of liver enzymes as well.^[16–18]^ The transaminitis in this case also resolved after cessation of the medication and administration of intravenous fluids.

Another component of *Triumeq* is abacavir, which is a nucleotide reverse transcriptase inhibitor. It carries relatively notorious adverse effect profile including hypersensitivity reaction, fever, nausea and diarrhea. Abacavir related hypersensitivity ranges from 3% to 6% and the median time to develop a hypersensitivity reaction is 11 days after starting the medication is started.^[19]^ The HLA-B5701 haplotype is listed as a significant risk factor for the development of hypersensitivity reactions, yet only 55% of HLA-B5701 positive individuals develop such reactions.^[20]^ Abacavir-induced hypersensitivity has been postulated to stem from mitochondrial toxicity, production of reactive metabolites, altered peptide repertoire presentation by the Major Histocompatibility Complex (MHC) I complexes and covalent haptenation of endogenous peptides.^[21]^ In our patient, HLA-B5701 was negative, thus hypersensitivity to abacavir would be unlikely. Abacavir induced rhabdomyolysis is rare, with a post marketing study done at Japan showing that this complication happened in 1 case out of 306 subjects analyzed^[21–23]^ and other reports stated that 6% of patients with abacavir hypersensitivity develop rhabdomyolysis.^[,6,7,19,21]^

Finally, *Triumeq* related postmarketing adverse reactions are reported, including rhabdomyolysis.^[24]^ Van Dam and Van Geffen^[25]^ described a case of young man who presented with *Triumeq* overdose and developed hypokalemia and lactic acidosis, which resolved after intravenous fluids and drug cessation without description of muscle toxicity or CK elevation. To the best of our knowledge, our patient did not overdose with *Triumeq* or had traditional risk factors for myositis/myopathy, yet developed acute rhabdomyolysis.^[26]^

## 4 Conclusion

Rhabdomyolysis can be a common finding in the HIV infected population, with a myriad of etiologies reported, including medication. While rare, rhabdomyolysis is a possible adverse reaction in patient receiving *Triumeq* and this case illustrates a suspected direct causal link with this medication. We consider that clinicians should be aware of this adverse event when managing patients with HIV infection using *Triumeq*. As mentioned above, rhabdomyolysis can be multifactorial in HIV infected patients, and *Triumeq* should be considered as an etiology in the appropriate setting. More data is warranted to elucidate the mechanism of rhabdomyolysis associated with *Triumeq*.

## Author contributions

**Supervision:** Paul Kelly.

**Writing – original draft:** Muhammad Saad, Fernando Casado-Castillo.

**Writing – review & editing:** Muhammad Saad, Paul Kelly, Fernando Casado-Castillo.
